# The Study of Tropical Medicine

**Published:** 1911-09-02

**Authors:** 


### THE STUDY OF TROPICAL MEDICINE.

*The London School of Tropical Medicine.*—The sessions, three a year, extend over three months each. The Colonial Office requires that men selected for posts in the tropics must have attended one such session, and, in addition to showing regularity in attendance, must have passed the examination at the end of the course. Failure to do so involves loss of the appointment. Other students may take the examination or not, as they choose, and, if successful, they are entitled to a certificate to that effect. The courses qualify, and are useful preparations, for the Diploma in Tropical Medicine and Hygiene granted by the University of Cambridge, and for the Diploma in Diseases and Hygiene of the Tropics granted by the Conjoint Board. The student is required to attend from 10 to 4 or 5 daily during the three months. In the wards of the Hospital that adjoins the school are always to be found a number of tropical cases newly arrived in the docks. These cases afford valuable instruction, as the majority have not been under treatment prior to their arrival in the wards.

*The Liverpool School of Tropical Medicine.*—This school is carried on in conjunction with the University of Liverpool and the Royal Southern Hospital, for the purposes of research and clinical teaching respectively. A special ward at the hospital has been set apart for the reception and treatment of tropical diseases, and facilities for investigation and study are given in the Johnston Laboratories of the University. The fee for a full course (lasting 13 weeks) is 13 guineas. A short course is also held during the month of June (fee 4 guineas).

---

The University of Edinburgh grants a diploma in tropical medicine and hygiene. The examinations are held in January and July. The fees for the first and any subsequent appearance are: Practical bacteriology, £1 1s.; diseases of tropical climates, £1 1s.; tropical hygiene, £1 1s.; tropical clinical medicine, £1 1s.; total, £4 4s.

The University will also grant a diploma in psychiatry. The fees for the first and second examinations are £5 5s. each. The first examination comprises anatomy, physiology, pathology, and bacteriology; the second examination, psychology (including experimental psychology), clinical neurology and psychiatry (systematic and clinical). The examinations will be held in March and June.

